# Effects of chronic allergic lung inflammation on gut microbiota and depression-like behavior in mice

**DOI:** 10.37349/eaa.2025.100978

**Published:** 2025-04-16

**Authors:** Akihiro Kanaya, Elvedin Luković, Charles Emala, Maya Mikami

**Affiliations:** 1 Department of Anesthesiology, Vagelos College of Physicians and Surgeons, Columbia University, New York, NY 10032, United States; 2 Department of Anesthesiology and Perioperative Medicine, Tohoku University School of Medicine, Sendai 980-8575, Japan

**Keywords:** Asthma, microbiome, gut-brain axis, allergy, depression

## Abstract

**Aim:**

Emerging epidemiological studies have reported a link between allergic diseases, including asthma, and depression. Evidently, the gut microbiota is involved in the pathogenesis of asthma and depression. Therefore, we investigated whether allergic lung inflammation in mice causes gut microbial dysbiosis, via the gut-brain axis, which is potentially associated with depression.

**Methods:**

Wild-type C57BL/6J female mice were sensitized with intranasal house dust mite (HDM) antigen or phosphate-buffered saline (PBS) for 6 weeks to induce chronic allergic lung inflammation. Sucrose preference tests were performed for assessing depression. Fecal samples were collected, and 16S ribosomal RNA gene sequencing was performed to detect differences in gut microbiota composition between the HDM and PBS groups. The distance calculation, clustering of operational taxonomic units, rarefaction analysis, and estimator calculation (α- and β-diversity) were performed.

**Results:**

There was a significant difference in β-diversity (Bray-Curtis dissimilarity, *F*-statistics = 6.16, *p* = 0.001) of the gut microbiota between HDM and PBS groups. However, there was no difference in the α-diversity. We observed multiple differentially abundant bacteria in the HDM and PBS groups. The order class Clostridia (*p* = 0.0036) and genus *Faecalibaculum* (*p* = 0.028) were more abundant in the HDM group, whereas the phylum Firmicutes (*p* = 0.037) and genera *Dubosiella* (*p* = 0.00024) and *Turicibacter* (*p* = 0.037) were more abundant in the PBS group. Notably, the relative abundance of some bacteria was correlated with the sucrose preference test results.

**Conclusions:**

Six weeks of intranasal HDM administration to mimic the chronic status of lung inflammation in asthma changed the gut microbiome in mice and was associated with depression-like behavioral changes.

## Introduction

Asthma is a critical global public health concern, which affected approximately 262 million people in 2019 [1] and causes approximately 455,000 deaths annually. Emerging epidemiological studies have shown that allergies and allergic diseases, including asthma, may be linked to depression [2, 3]. Our previous study demonstrated a correlation between allergic lung inflammation and alterations in brain inflammation suggested by inflammatory markers measured in the lung, serum, and the brain, leading to behavioral changes indicating depression and impaired spatial memory [4]. Nevertheless, the mechanism(s) underlying asthma-related depression are partially understood and warrant further investigation.

The human body hosts an array of indigenous microorganisms that collectively form the microbiota, including bacteria, archaea, fungi, and viruses. This microbiota is present on the skin and mucous membranes throughout the body, including the oral and nasal cavities, lungs, esophagus, small and large intestine, uterus, vagina, bladder, and ureters. The intestinal tract, particularly, provides a conducive environment for bacterial growth owing to its eutrophic and anaerobic conditions, with approximately 1,000 species or 100 trillion bacteria maintaining a higher density than the external environment [5]. Consequently, the bacterial flora is crucial for human homeostasis, and dysbiosis can influence disease susceptibility. Several reports have highlighted the connection between intestinal flora and various diseases, including allergies, rheumatism, cancer, multiple sclerosis, Parkinson’s disease, inflammatory bowel disease, and depression [6–8]. Depression is frequently observed in patients with asthma; additionally, the prevalence of depression among patients with asthma is 11–13% worldwide [3, 9]. Reportedly, the gut microbiota is (partially) involved in the development of asthma and depression [10, 11]. However, the precise role of the gut microbiota in asthma-related depression is yet to be elucidated.

Considerably, we used 16S rRNA sequencing to assess and compare the composition of the gut microbiota in mice with and without allergic lung inflammation induced by house dust mite (HDM) exposure, a well-established mouse model of allergic asthma. In this cohort, we previously demonstrated enhanced lung inflammation and anhedonia [4]. Herein, we investigated the hypothesis that allergic lung inflammation in mice causes gut microbial dysbiosis, which may further contribute to depression through the gut-brain axis.

## Materials and methods

### Animals

**Figure 1.**
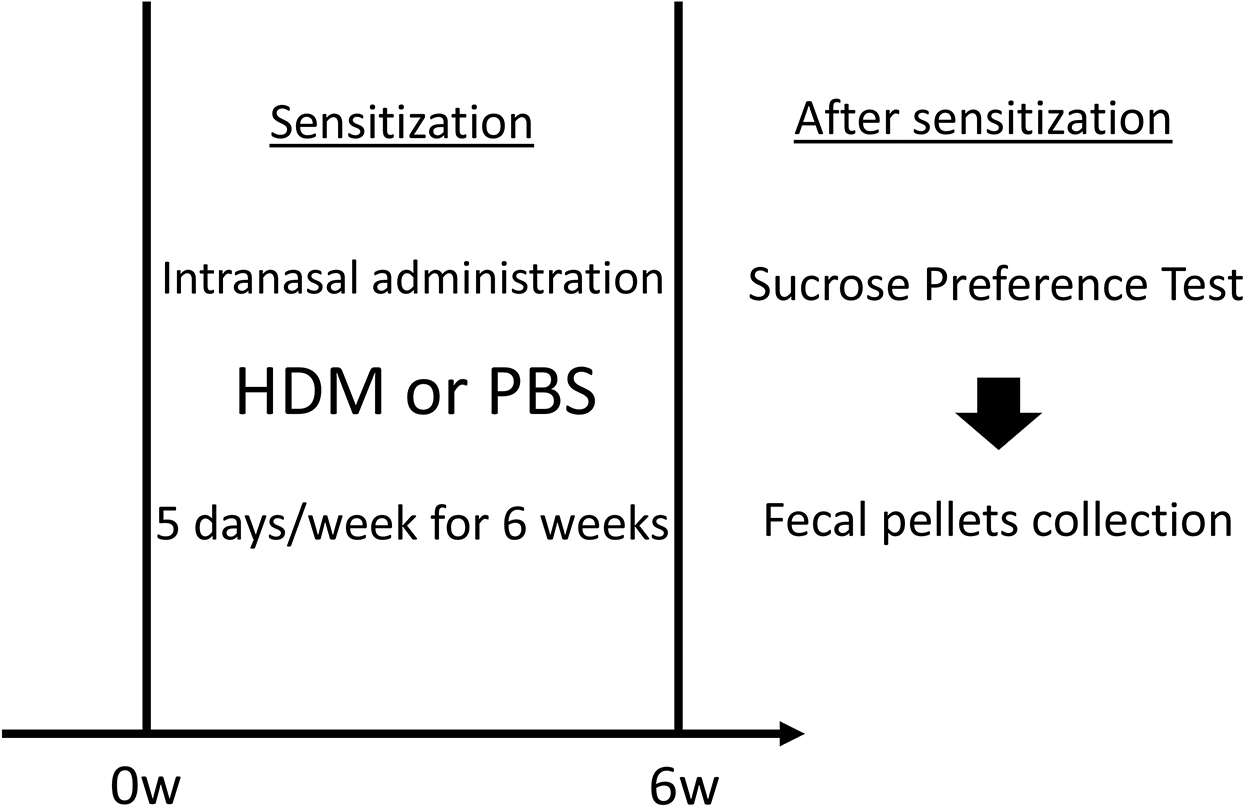
Schematic of the timeline of experiments. Mice were sensitized by the intranasal administration of HDM or control PBS, 5 days/week for 6 weeks. We administered 30 μg in 25 μL of HDM intranasally under isoflurane anesthesia. After sensitization, mice underwent sucrose preference testing to assess depressive-like behavior, followed by the collection of fecal pellets. In the test, the mice were provided two bottles in a single cage: one containing fresh 1% sucrose solution and another containing fresh tap water. HDM: house dust mite; PBS: phosphate-buffered saline

All procedures involving animals were approved by the Columbia University Institutional Animal Care and Use Committee. The 8- to 10-week-old C57BL/6J female mice were purchased from the Jackson Laboratory (Bar Harbor, ME) and allowed to acclimate to the vivarium before experiments. Initially, all mice were housed in groups of four mice per cage. Only female mice were used herein because we observed constant fighting among group-housed male mice in a previous pilot study, which potentially affected their behavior and depression symptoms. The timeline of the experiments is shown in Figure 1. HDM extracts were purchased from Stallergenes Greer (Lenoir, NC) and reconstituted in endotoxin-free phosphate-buffered saline (PBS, MilliporeSigma, Burlington, MA). We administered 30 μg in 25 μL of HDM intranasally using 2–5% isoflurane anesthesia once daily for 5 days/week for 6 weeks. The PBS control mice received an equivalent volume of PBS via intranasal administration under the same anesthesia protocol.

### Sucrose preference test

After 6 weeks of intranasal sensitization, we performed a sucrose preference test to investigate depression in mice, as previously described in this cohort of mice [4]. The two-bottle choice test is a standard assay for testing anhedonia and depression-like behaviors. During the baseline exposure period, each mouse had access to regular chow and two bottles (Nalgene 50 mL centrifuge tubes, Nalge Nunc International Corporation, Rochester, NY) in their home cage. One bottle contained a 1% sucrose solution, and another contained regular tap water. This arrangement was maintained for 48 h, with the position of the two bottles changed every 24 h to avoid bias related to bottle location. Subsequently, each mouse was moved to its cage and provided only regular tap water (from a standard water bottle) and regular chow for the next 24 h. On the testing day (from 8 pm to 8 am), the mice were again provided two bottles in a single cage: one containing fresh 1% sucrose solution and another containing fresh tap water. At the end of the test, the bottles were collected, and their contents were weighed. During the two-bottle choice test, regular bedding was removed and replaced with a few pieces of paper towels to detect potential leakage. Nalgene tubes were washed with fragrance-free detergent (Seventh Generation, Burlington, VT) and air-dried after each use.

### 16S rRNA analysis of fecal samples

Following the completion of all behavioral tests, mice were anesthetized with an intraperitoneal injection of pentobarbital sodium (100 mg/kg), and fresh fecal pellets were collected. The samples were placed in 1.5 mL tubes and stored at −80°C. Subsequently, the frozen samples were sent to the Center for Metagenomics and Microbiome Research at Baylor College of Medicine, where 16S rRNA gene sequence libraries were generated from the V4 primer region using an Illumina MiSeq platform [12]. DNA was extracted using a PowerMag Soil DNA Isolation Kit (formerly from MO BIO Laboratories, now part of QIAGEN). Data analysis, including distance calculation, operational taxonomic unit (OTU) clustering, rarefaction analysis, and estimator calculation, was performed using Agile Toolkit for Incisive Microbial Analysis, a tool developed by the Center for Metagenomics and Microbiome Research at Baylor College of Medicine. Resulting reads were clustered into OTUs at a similarity cutoff of 97% via the UPARSE algorithm [13] using an in-house stepwise approach that includes chimera filtering. The final output was run against the Genomes OnLine Database (GOLD) [14, 15]. Then, the OTU centroids were mapped against an optimized version of the latest current SILVA Database [16] containing only sequences from the appropriate V region of the 16S rRNA gene to determine taxonomies, specifying the identity threshold at 97%. We aimed for an average per sample yield of 25,000 read pairs.

### Statistical analysis

The data were normalized by rarefying all samples to 18,453 reads per sample, which was the lowest number of reads produced by any sample in the project. Continuous variables were presented as mean ± standard error. The Mann-Whitney U test was used to assess differences between the two groups. Furthermore, correlation analysis was conducted using Pearson’s product-moment coefficient. *p* < 0.05 was considered statistically significant. *R* (version 4.3.1) was used for statistical computing.

## Results

### α- and β-diversity comparison of murine gut microbiota between the PBS and HDM groups

**Figure 2.**
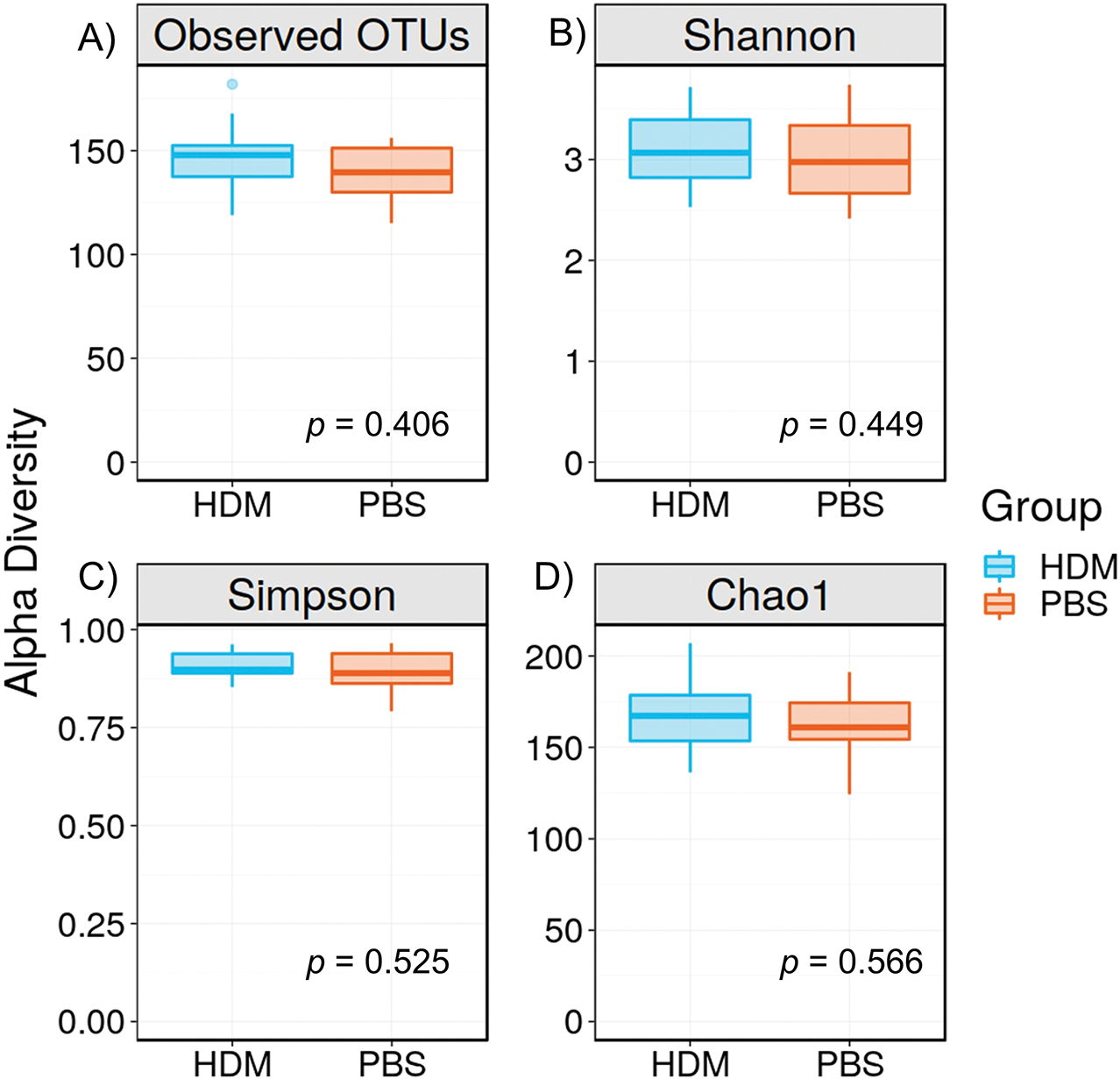
α-diversity of the gut microbiota. **A**) OTUs, **B**) Shannon index, **C**) Simpson index, and **D**) Chao1 index. There was no significant difference in the OTUs (*p* = 0.406, Mann-Whitney), Shannon Index (*p* = 0.449, Mann-Whitney), Simpson Index (*p* = 0.525, Mann-Whitney), and Chao1 (*p* = 0.566, Mann-Whitney) between the HDM sensitization and PBS control groups, suggesting that the number of species, richness, and distribution were not different between groups. Data is presented as mean ± standard error. OTUs: operational taxonomic units; HDM: house dust mite; PBS: phosphate-buffered saline

α-diversity measures the richness (number) and distribution (evenness or abundance) of bacterial species within a specific community or habitat (HDM and PBS in this case). We evaluated α-diversity using the Shannon, Simpson, and Chao1 indices of gut microbiota. They are frequently used to measure species diversity in a given community. When we compared α-diversity of HDM and PBS cohorts, no considerable difference was found with either the Shannon, Simpson, or Chao1 tests, indicating that the two populations retain similar within-sample diversity (Figure 2). The Simpson index for the HDM and PBS control groups was nearly 1, suggesting that all our samples were diverse. Our mice were housed in groups of four, and those housed in the same cage tend to share similar gut microbiota owing to mixing by coprophagia. Therefore, we tested the cage effect by performing an analysis to compare the α-diversity categorized by the cage and found no difference (Figure S1).

**Figure 3.**
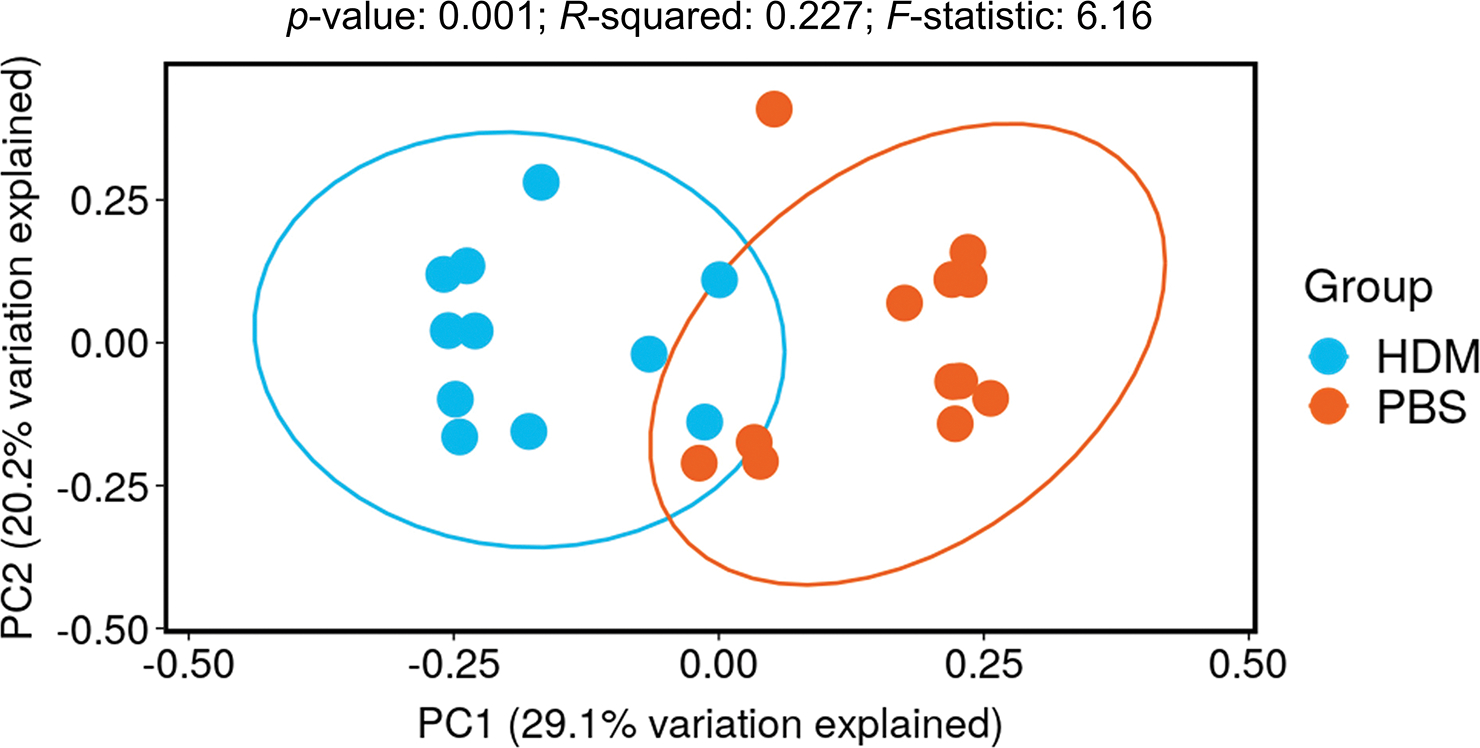
PCoA plot of Bray-Curtis dissimilarity (β-diversity). Dots represent individual mice in the PBS control (orange) or HDM (blue) groups: the X and Y axes represent the degree of major influence in percentage, in which the closer the distance, the higher the similarity. The HDM group had a significantly decreased PC1 score compared with the PBS control group, *p* < 0.05. HDM: house dust mite; PBS: phosphate-buffered saline; PC: principal component; PCoA: principal coordinates analysis

In contrast, β-diversity is a measure of similarity/dissimilarity among groups (HDM vs. PBS in this case). Principal coordinate analysis (PCoA) plots of Bray-Curtis dissimilarity (β-diversity) revealed a distinct separation between the HDM and PBS groups (Figure 3), suggesting that there are considerable differences in the gut microbiota composition between these two groups.

### Gut microbiota composition at the phylum, class, order, family, and genus levels in PBS and HDM mice

**Figure 4.**
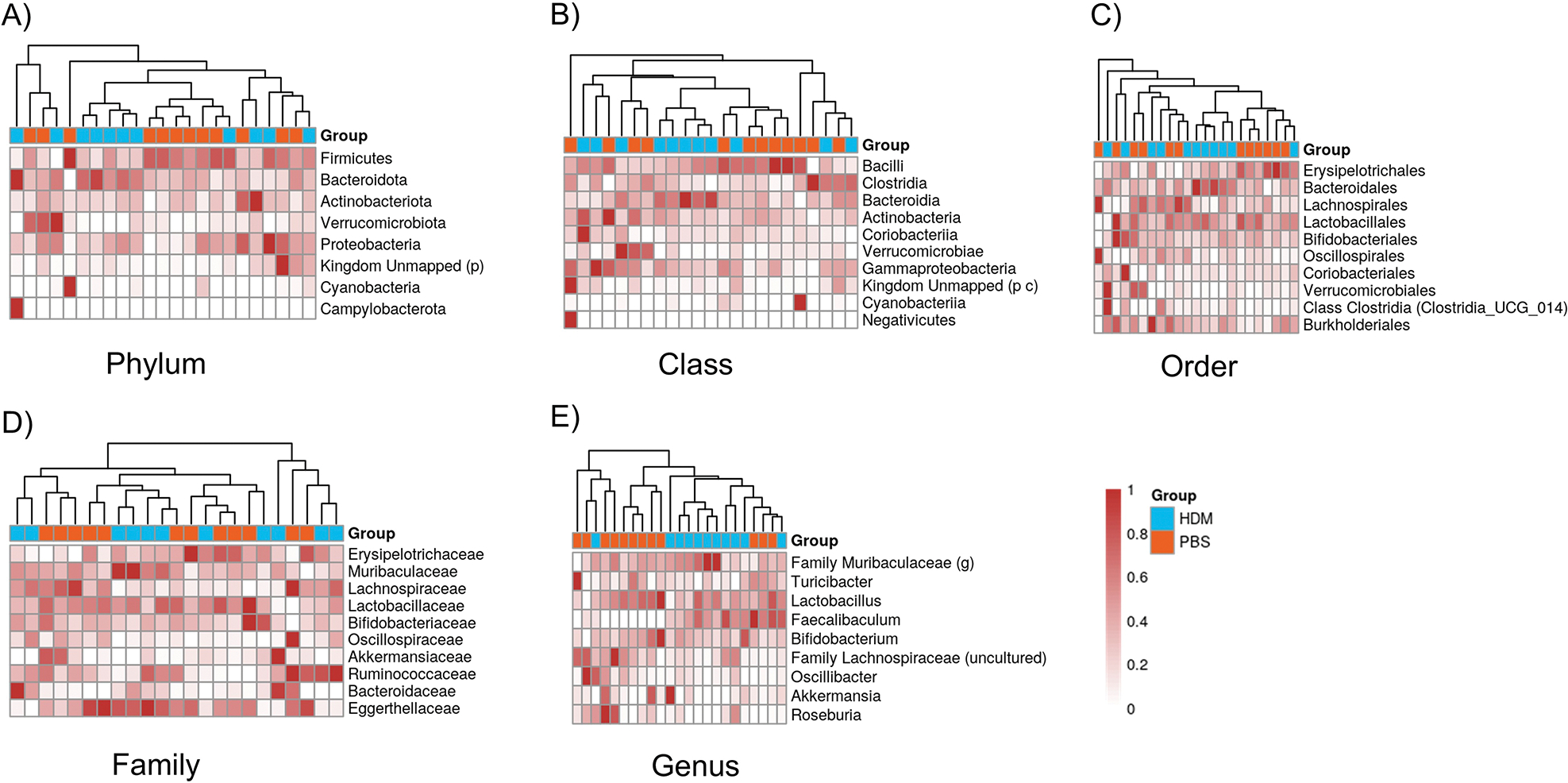
Heatmaps of the composition of gut bacteria between the PBS and HDM groups at different taxonomic ranks. **A**) Phylum, **B**) class, **C**) order, **D**) family, and **E**) genus levels. The X-axis shows the PBS control (orange) and HDM (blue) samples, and the Y-axis shows the bacterial groups. Legends at the right indicate the color key representing relative abundance, with light and dense colors indicating relatively low and high abundances, respectively. Heatmaps typically show that samples cluster by treatment group (PBS or HDM), and they are separated. HDM: house dust mite; PBS: phosphate-buffered saline

A heat map of the Bray-Curtis dissimilarity distance, displaying the composition of gut microbiota at the phylum, class, order, family, and genus levels in PBS and HDM groups, is completely different (Figure 4). For example, in Figure 4A, showing the composition at the phylum level, PBS and HDM groups (in orange and blue, respectively) generally form separate clusters, indicating that although the two groups display similar α-diversity (Shannon/Simpson/Chao1), they are different in composition at many taxonomic ranks.

### Alterations in the gut microbiota composition between PBS and HDM mice

**Figure 5.**
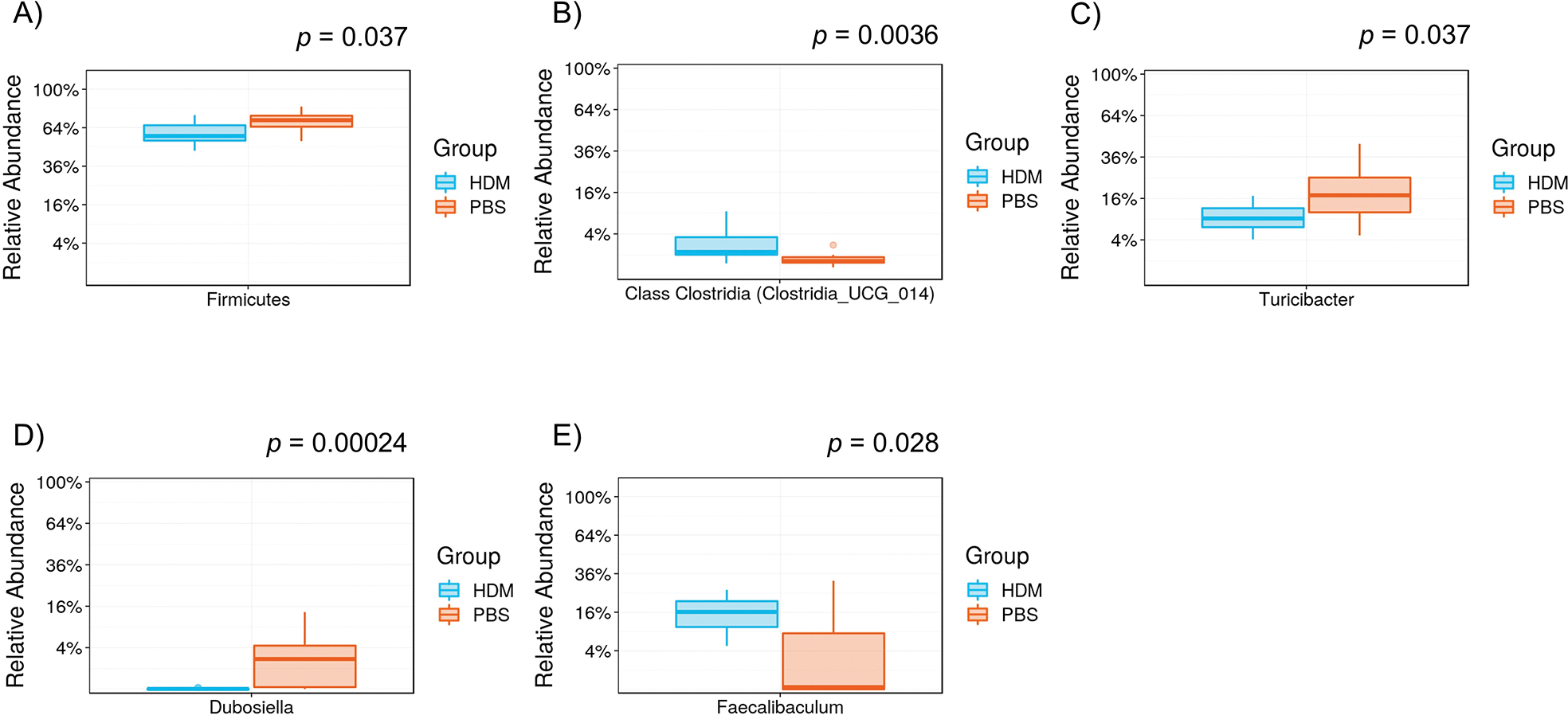
Differential levels of the gut bacterium between PBS and HDM groups. Relative abundance of **A**) phylum Firmicutes, **B**) order class Clostridia, **C**) genus *Turicibacter*, **D**) genus *Dubosiella*, and **E**) genus *Faecalibaculum*: the order class Clostridia (*p* = 0.0036, Mann-Whitney) and genus *Faecalibaculum* (*p* = 0.028) were more abundant in the HDM group, whereas the phylum Firmicutes (*p* = 0.037), genus *Dubosiella* (*p* = 0.00024), and genus *Faecalibaculum* (*p* = 0.028) were more abundant in the PBS control. HDM: house dust mite; PBS: phosphate-buffered saline

We used 16S rRNA gene sequencing to determine the differences in the composition of gut microbiota between the PBS and HDM mice. Considerable alternations were noted in the fecal samples of mice between the two groups (Figure 5A–E). Specifically, five gut bacteria at three phylogenetic levels (phylum, order, and genus) differed considerably. At the order level, the Clostridia class (Figure 5B, *p* = 0.0036) and at the genus level, *Faecalibaculum* (Figure 5E, *p* = 0.028) were more abundant in the HDM group, whereas the phylum Firmicutes (Figure 5A, *p* = 0.037), the genus *Turicibacter* (Figure 5C, *p* = 0.037), and *Dubosiella* (Figure 5D, *p* = 0.00024) were more abundant in the PBS group.

### Correlation analysis between sucrose preference rate and the abundance of gut bacteria

**Figure 6.**
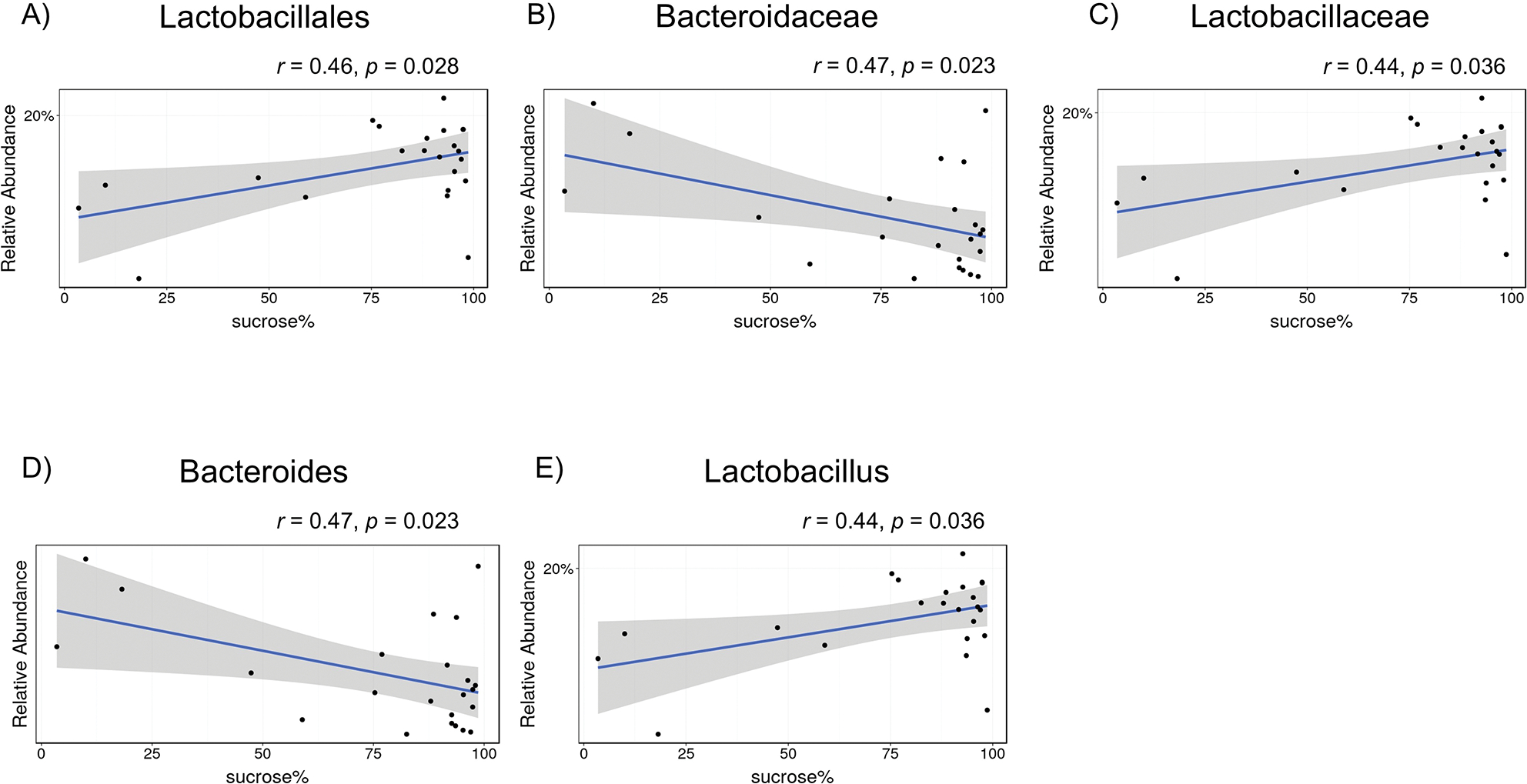
Correlations between sucrose preference rate and the composition of gut bacteria among all mice (phosphate-buffered saline and house dust mite). **A**) Order Lactobacillales, **B**) family *Bacteroidaceae*, **C**) family *Lactobacillaceae*, **D**) genus *Bacteroides*, and **E**) genus *Lactobacillus*. The X-axis shows the sucrose water preference over plain water consumption expressed as a percentage. Each dot represents a single mouse

A sucrose preference test was conducted to assess the association between mouse depression and gut microbiota. Reportedly, the order Lactobacillales (Figure 6A, *r* = 0.46, *p* = 0.028), family *Lactobacillaceae* (Figure 6C, *r* = 0.44, *p* = 0.036), and genus *Lactobacillus* (Figure 6E, *r* = 0.44, *p* = 0.036) were positively associated with sucrose preference tests, whereas the family *Bacteroidaceae* (Figure 6B, *r* = 0.47, *p* = 0.023) and genus *Bacteroides* (Figure 6D, *r* = 0.47, *p* = 0.023) were negatively associated with sucrose preference tests. The five gut bacteria at three phylogenetic levels, in which relatively abundant differences were observed between the two groups, did not show statistically significant correlations with sucrose preference (data not shown).

## Discussion

Herein, we observed considerable differences in the gut microbial β-diversity between mice subjected to allergic lung inflammation with intranasal HDM sensitization for 6 weeks compared with control mice that received PBS. Further analysis revealed considerable differences in the composition of gut microbiota between HDM and PBS mice at three taxonomic ranks (phylum, order, and genus), involving five gut bacterial cohorts (phylum Firmicutes, order class Clostridia, genus *Faecalibaculum*, genus *Turicibacter*, and genus *Dubosiella*). Moreover, the abundance of several gut bacteria was associated with a preference for drinking sucrose water, a standard measure of anhedonia/depression-like behavior in mice.

Our previous study reported lung histopathological analysis, bronchoalveolar lavage (BAL) cell counts, BAL protein concentration measurements, and cytokine measurements, confirming the effect of HDM on lung inflammation [4]. This study is novel because it reports the alteration of gut microbiota and its association with depression-like behavior using a mouse model of asthma. For example, previous studies reported stool microbial composition in children with mite-sensitized asthma and rhinitis [17]; however, associated symptoms in patients were not investigated. Another study utilized an ovalbumin-induced allergic airway inflammation model to investigate prior exposure to an environmental bacteria and subsequent changes in the gastrointestinal microbiome [18]; however, the model used to induce allergic airway inflammation was different from this study, and their investigation focused on bacterial exposure effect on subsequent microbiome alteration, which is also different from this experimental design.

Regarding α-diversity, which assesses the diversity of species and bacteria within a community, no considerable differences were observed between the PBS and HDM groups (Figure 2), suggesting that the overall number and abundance of bacterial taxa in the gut microbiota were similar between the groups. However, α-diversity alone does not report the specific composition or relative abundance of microbial taxa [19]; rather, it is a measure of the richness and evenness of a particular sample. In contrast, the β-diversity analysis conducted using PCoA revealed a considerable separation between the HDM and PBS groups (Figure 3), indicating distinct differences in the overall composition of the gut microbiota between the two groups [20]. The observed separation in the analysis suggests that the gut microbial community responded differently to HDM sensitization.

Further exploration of the gut microbiota composition at different taxonomic levels (phylum, class, order, family, and genus) revealed specific alterations between the PBS and HDM groups. The genus *Faecalibaculum* was abundant in the HDM group, whereas the phylum Firmicutes and genera *Dubosiella* and *Turicibacter* were abundant in the PBS group. Although limited studies have reported differences in the gut microbiome between the HDM and control groups in mice, these differences in microbial taxa suggest that intranasal exposure to HDM and the development of chronic allergic lung inflammation might have influenced the relative abundance of specific bacteria within the gut microbiota in mice.

To investigate the potential association between depression-like behavior and gut microbiota, a correlation analysis with sucrose preference tests was conducted. Although animal models are not a perfect representation of human depression, several behaviors observed in mouse models have been used, including reduced activity, social withdrawal, despair, and anhedonia [4]. The sucrose preference test is often used to measure anhedonia, which is considered a symptom of depression in mice. Our analysis revealed some intriguing associations. Elevated levels of the order Lactobacillales, family *Lactobacillaceae*, and genus *Lactobacillus* were positively correlated with sucrose preference tests, suggesting that the absence of these bacterial groups may be associated with depressive-like behaviors. However, because of the variance in the sucrose test, we cannot exclude other factors that potentially contribute to the result of this behavior test. Several studies have demonstrated the beneficial effects of *Lactobacillus* on mice depression evaluated by forced swim, sucrose preference, and nest-building tests, which align with our findings [21–23]. *Lactobacillus* produces gamma-aminobutyric acid, thereby leading to cognitive enhancement via the vagus nerve [24]. In addition, the previous review explores the complex interactions between the gut-dwelling *Lactobacillus* and immune components in distant organs to promote host health, by strong involvement in the gut-brain and gut-lung axes [25]. Moreover, the ability of *Lactobacillus* to reduce susceptibility to allergic asthma has been reported in several mouse models of allergic lung inflammation [26, 27]. In human studies comparing stool microbial diversity in adults, the significant difference between patients with asthma and healthy controls in *Lactobacillus* was not found, potentially because of variations across diverse contexts including age, diet, environmental influences, history of infections, and antibiotics use [28]. Nevertheless, its potential beneficial effect on depression-like behavior in allergic lung inflammation requires further investigation, utilizing simpler, controlled animal models.

Conversely, increased levels of the family *Bacteroidaceae* and genus *Bacteroides* showed negative correlations with sucrose preference tests, implying a potential risk of increased depression-like behavior with increased abundance of *Bacteroidaceae* and *Bacteroides*. Reportedly, Bacteroidetes showed higher relative abundances in the homes of patients with atopy than those without it, indicating a potentially harmful association [29]. Atopy remains a strong, identifiable predisposing factor for developing allergic asthma, with approximately 25–30% of patients with atopy developing asthma [30]. Asthma control and severity are associated with symptoms of depression, and a prospective cohort study reported that 25% of participants with severe asthma expressed depression compared with 9% of the nonsevere asthma population [31]. Previous observational studies comparing patients with depressive disorder and healthy controls found an increased abundance of the phylum Bacteroidetes in the depressive disorder group [32–34]. Herein, our findings suggest that an increased abundance of *Bacteroidaceae* and *Bacteroides* is associated with decreased sucrose preference in mice, which agrees with studies reported previously.

The findings of this study help understand the relationship between gut microbiota composition and depression-like behavior. The observed differences in β-diversity and alterations at various taxonomic levels suggest that changes in the gut microbiota are potentially influenced by HDM sensitization and may impact behavioral health outcomes. Positive and negative associations between specific microbial taxa and sucrose preference tests indicate that certain bacteria may be involved in modulating depressive-like behaviors, suggesting that the gut-brain axis may be crucial for the asthma population [35]. It would be interesting to further study microbial metabolite changes in HDM-sensitized mice that may contribute to the gut-brain axis alterations.

Our results are based on relative taxonomic abundances (limited to the genus level); therefore, an increase or decrease in the relative abundance of a bacterium does not mean that a bacterium is actually increasing or decreasing in absolute amount. In addition, our findings are based on an animal model and may not directly translate to humans. Further research is required to elucidate the mechanisms underlying the observed associations and investigate the potential therapeutic implications of manipulating the gut microbiota by, for example, probiotic supplementation to increase beneficial taxa and/or microbiota transplantation, for behavioral health interventions in humans.

## Conclusion

Six weeks of intranasal HDM administration in mice aimed at replicating the chronic lung inflammatory state found in asthma and led to several changes in the gut microbiota. Specific alterations in the composition of the gut microbiota were associated with sucrose preference rates, suggesting an association between asthma-related depression and the microbiota. The lung-gut-brain axis involving chronic inflammation and microbiota may play pivotal roles in asthma-related depression in mice.

## Supplementary Material

Supplemental Figure 1

Supplementary materials

The supplementary Figure S1 for this article is available at: https://www.explorationpub.com/uploads/Article/file/100978_sup_1.pdf.

## Data Availability

The data that support the findings of this study are available from the corresponding author upon reasonable request.

## Abbreviations

BAL: bronchoalveolar lavage
HDM: house dust mite
OTUs: operational taxonomic units
PBS: phosphate-buffered saline
PCoA: principal coordinate analysis
