# Supplementary figures and images for: Effects of chronic allergic lung inflammation on gut microbiota and depression-like behavior in mice
Source: Explor Asthma Allergy. Author manuscript; Available in PMC 2026 Jan 9. (PMC12781662; doi:10.37349/eaa.2025.100978)

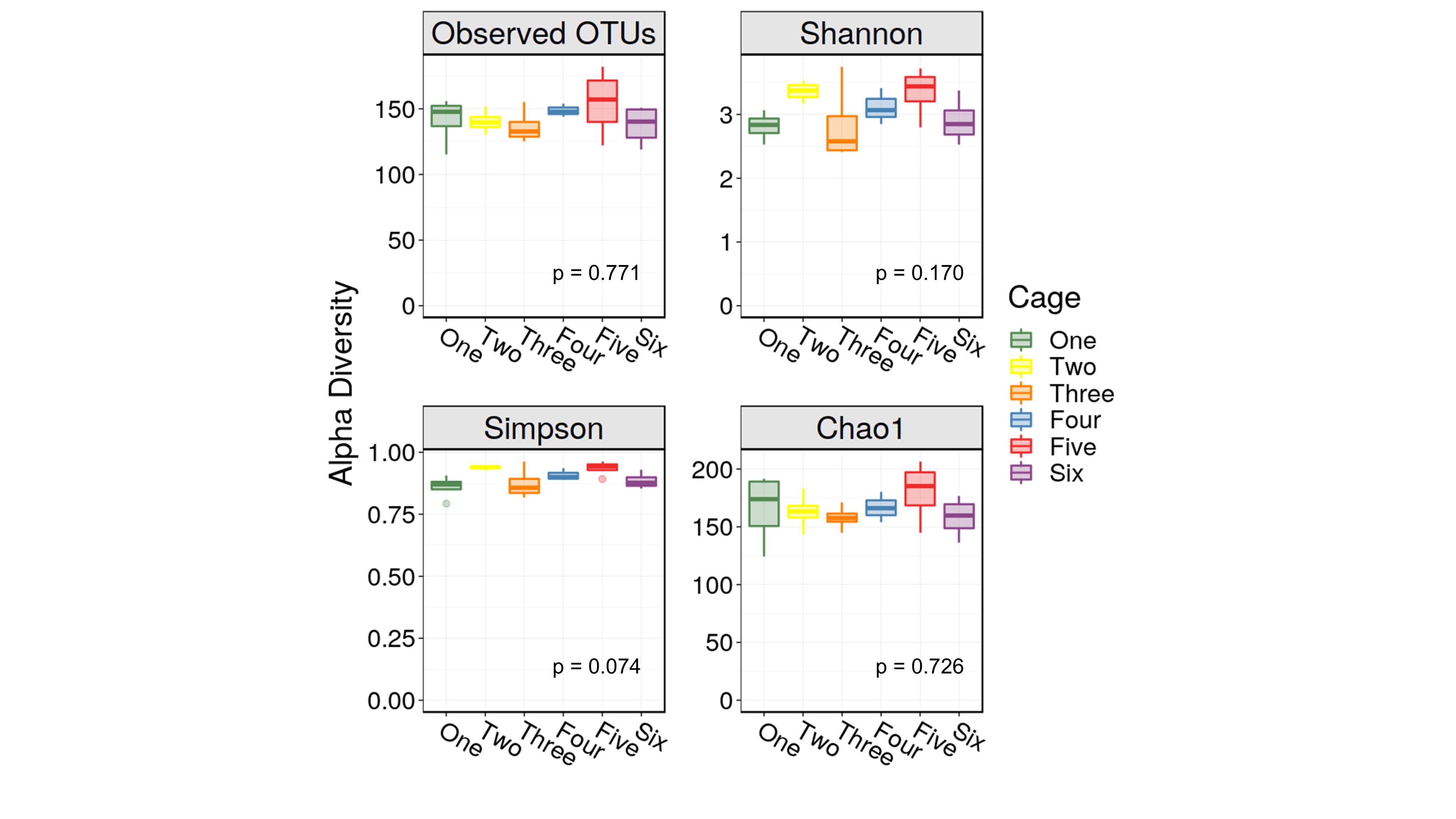

Supplement: Supplemental Figure 1 [file NIHMS2114106-supplement-Supplemental_Figure_1.jpg]
